# The Institutional Library

**Published:** 1921-08-27

**Authors:** 


### THE INSTITUTIONAL LIBRARY.

**Diseases of the Ear, Nose, and Throat in Childhood.** By Douglas Guthrie, M.D., F.R.C.S., Surgeon to the Ear and Throat Department, Royal Hospital for Sick Children, Edinburgh; Surgeon to the Edinburgh Ear and Throat Infirmary. (London: A. & C. Black. 1921. Pp. 88. Price 5s.)

In this little book an endeavour is made, so says the author in an introductory note, to supply some practical information regarding the common diseases of the ear, nose, and throat in early life to general practitioners, medical inspectors of schools, and superintendents of child-welfare centres, and it is also hoped that it may be intelligible and helpful to nurses and social workers. General practitioners and medical inspectors are expected to have a more thorough knowledge of these subjects than is conveyed in this short work, which, however, is clearly written and should be of use to nurses and child-welfare workers. It will do a good work if it diffuses a knowledge of the enormous amount of damage caused to the population by the prevalent neglect of the common diseases of the ear and throat.

**Materia Medica and Pharmacy.** By Reginald R. Bennett, B.Sc. (Lond.), F.I.C. Fourth edition. (London: H. K. Lewis & Co., Ltd. Pp. xxiii and 264, fcp. 8vo. Price 7s. 6d. net.)

The present edition of this useful little book contains fuller information than its predecessors, both in respect of the actions of drugs and the pathological conditions for which their various preparations are prescribed. Dosage is given in both metric and imperial systems; but, as the author points out, the relation between the two in any given instance is of approximate accuracy only. The book appears to be complete, and is concise and reliable. There is a useful appendix on "Incompatibility," and a sufficient index. In general appearance it is neat, and, altogether, should maintain the reputation of the previous editions.

**Tuberculosis: its Prevention and Home Treatment.** By H. Hyslop Thompson, M.D., D.P.H. Second edition. Sm. 8vo. Pp. 99. Oxford Medical Publications. (London: Henry Frowde and Hodder and Stoughton. N.D. Price 4s. net.)

This is a good little manual, its only fault that the title is too ambitious; it should have been "*Pulmonary* Tuberculosis," etc. The book is good because the author has had practical experience in charge of a sanatorium, and therefore knows which points to stress. He knows what is pyrexia, which is more than half the authors on tuberculosis do, and when to send the patient to bed. It is a saddening thought how much human suffering would have been saved if that simple bit of knowledge had been prevalent in this country years ago. We need an Oxford medical publication on surgical tuberculosis, and the man to write it is Sir Henry Gauvain. It would have far fewer rivals than the present publication, which, however, we have no fear in recommending in preference to any others.

